# Supplementary material for: Anemia during pregnancy and adverse maternal outcomes in Georgia–A birth registry-based cohort study
Source: PLoS One. 2025 Jan 30;20(1):e0294832. doi: 10.1371/journal.pone.0294832 (PMC11781653; doi:10.1371/journal.pone.0294832)
Supplement: S1 File — (DOCX) [file pone.0294832.s001.docx]

**Supplementary file 1**

In this study, we used direct acyclic graphs (DAGs) to identify confounding factors for the presumed causal relationship between anemia and maternal post-delivery intensive care unit (ICU) admission.

DAGs are useful tools for conceptualizing frameworks and visualizing assumed relationships between exposures, outcomes, and covariates [1]. In the figure below, variables indicated with pink circles are identified as confounders, and blue circles indicate mediating variables. We included the identified confounding factors in the regression analysis to estimate the total effect of the exposure on the outcome.

The assumptions for the causal effect of anemia on post-delivery ICU admission are presented in supplementary figure 1. We assumed that age, education, BMI, twin/triplet pregnancy, and bleeding during pregnancy impacted the possibility of anemia during pregnancy. Further, cesarean section (CS) delivery, bleeding during pregnancy, BMI, and post-partum hemorrhage (PPH) are risk factors for ICU admission [2–6]. Several other factors, such as parity and placenta previa, are risk factors for anemia, ICU admission, or both. Further, many risk factors for anemia directly or indirectly increase the risk of ICU admission. Thus, the minimal sufficient set of variables to be included in the model to control for confounding factors and estimate the total effect of anemia-related infection on post-delivery ICU transfer includes age, BMI at the first ANC visit, bleeding during pregnancy, cesarean section (CS), and parity.

*Supplementary Figure 1: A directed acyclic graph depicting the relationship between anemia in pregnancy, number of covariates, and post-delivery intensive care unit (ICU) admission*


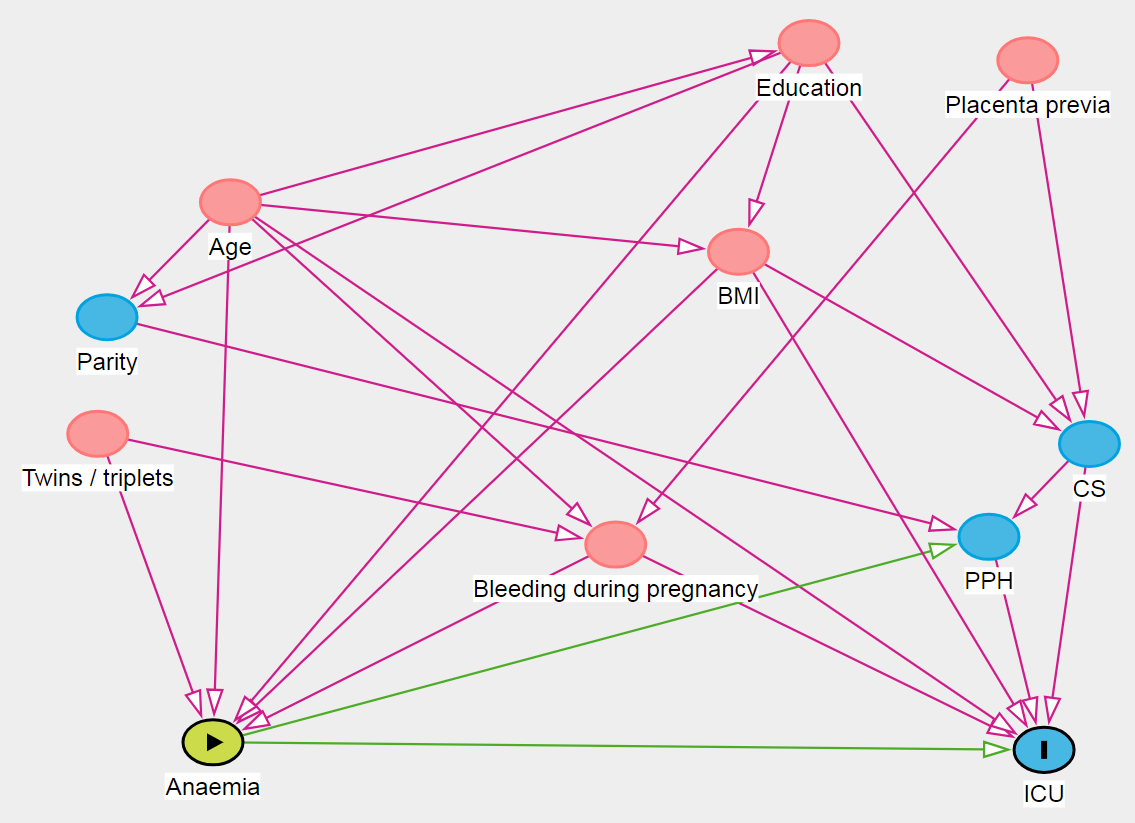


**References:**

1. Tennant PWG, Murray EJ, Arnold KF, Berrie L, Fox MP, Gadd SC, et al. Use of directed acyclic graphs (DAGs) to identify confounders in applied health research: review and recommendations. Int J Epidemiol. 2021;50: 620-632. doi: [10.1093/ije/dyaa213](https://doi.org/10.1093/ije/dyaa213).

2. World Health Organization. WHO recommendations on antenatal care for a positive pregnancy experience. Summary. 2018. Available from: <https://apps.who.int/iris/bitstream/handle/10665/259947/WHO-RHR-18.02-eng.pdf>.

3. World Health Organization. Maternal, infant and young child nutrition. Comprehensive implementation plan on maternal, infant and young child nutrition: biennial report; 2021.

4. Auerbach M. Anemia in Pregnancy; 2023. Available from: www.uptodate.com.

5. Barut A, Mohamud DO. The association of maternal anaemia with adverse maternal and foetal outcomes in Somali women: a prospective study. BMC Womens Health. 2023;23: 193. doi: [10.1186/s12905-023-02382-4](https://doi.org/10.1186/s12905-023-02382-4).

6. WOMAN-2 trial collaborators. Electronic address: woman2@lshtm.ac.uk, WOMAN-2 trial collaborators. Maternal anaemia and the risk of postpartum haemorrhage: a cohort analysis of data from the WOMAN-2 trial. Lancet Glob Health. 2023;11: e1249–e1259-e59. Epub 20230627. doi: [10.1016/S2214-109X(23)00245-0](https://doi.org/10.1016/s2214-109x(23)00245-0).
