# Supplementary material for: Anemia during pregnancy and adverse maternal outcomes in Georgia–A birth registry-based cohort study
Source: PLoS One. 2025 Jan 30;20(1):e0294832. doi: 10.1371/journal.pone.0294832 (PMC11781653; doi:10.1371/journal.pone.0294832)
Supplement: S2 File — (DOCX) [file pone.0294832.s002.docx]

**Supplementary file 2**

In this study, we used direct acyclic graphs (DAGs) to identify confounding factors for the presumed causal relationship between anemia and preterm delivery.

DAGs are useful tools for conceptualizing frameworks and visualizing the assumed relationships among exposures, outcomes, and covariates [1]. In the figures below, variables indicated with pink circles are identified as confounders, and blue circles indicate mediating variables. The identified confounding factors were included in the regression analysis to estimate the total effect of exposure on the outcome.

Assumptions for the presumed causal effect of anemia on preterm delivery are presented in the DAG in supplementary figure 2. We assumed that age, education, BMI at the first ANC visit, twin/triplet pregnancy, and bleeding during pregnancy impact the possibility of anemia in pregnancy [2-6], and that age, BMI, twin/tripletpregnancy, bleeding during pregnancy, smoking, and placenta previa increase the risk of preterm delivery. Several risk factors for anemia also directly or indirectly increase the risk of preterm delivery. Hence, the minimal sufficient set of variables to include in models to control for confounding factors and estimate the total effect of anemia on preterm delivery includes age, education, BMI at the first ANC visit, bleeding during pregnancy, and plural pregnancy (twins/triplets).

Supplementary Figure 2: A directed acyclic graph depicting the relationship between anemia in pregnancy, covariates, and preterm delivery


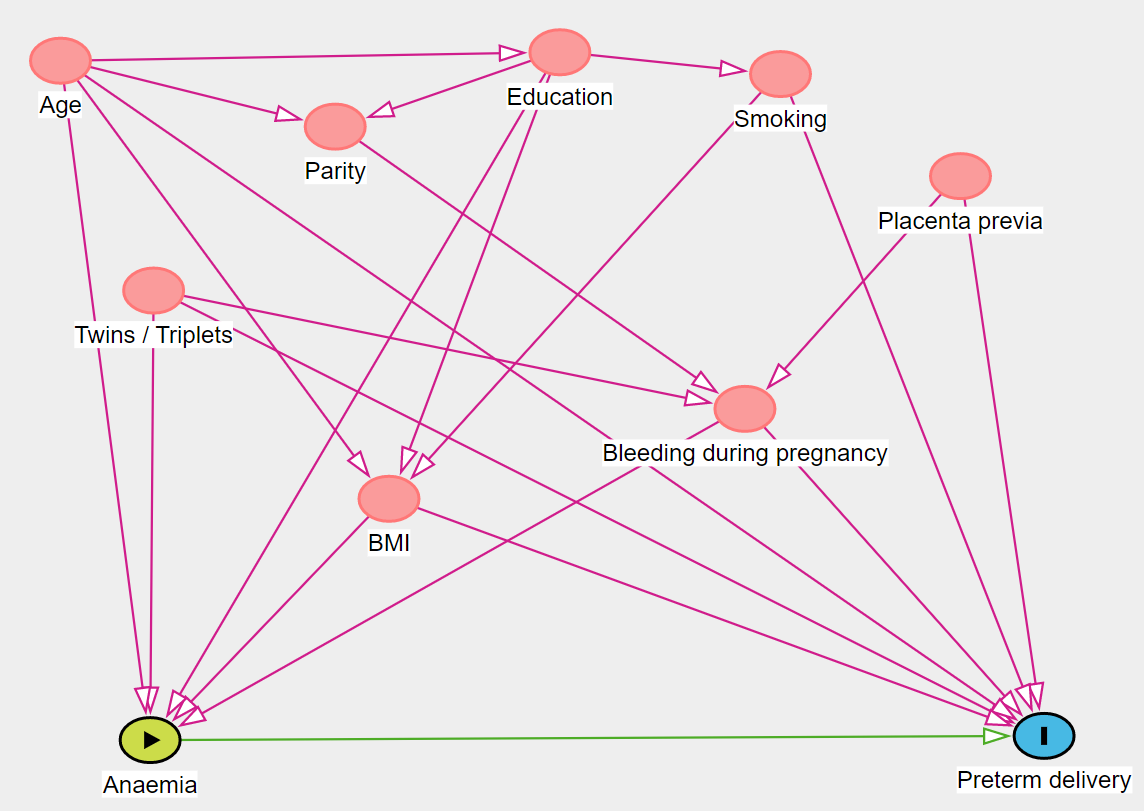


1. Tennant PWG, Murray EJ, Arnold KF, Berrie L, Fox MP, Gadd SC, et al. Use of directed acyclic graphs (DAGs) to identify confounders in applied health research: review and recommendations. Int J Epidemiol. 2021;50: 620-632. doi: [10.1093/ije/dyaa213](https://doi.org/10.1093/ije/dyaa213).

2. World Health Organization. WHO recommendations on antenatal care for a positive pregnancy experience. Summary. 2018. Available from: <https://apps.who.int/iris/bitstream/handle/10665/259947/WHO-RHR-18.02-eng.pdf>.

3. World Health Organization. Maternal, infant and young child nutrition. Comprehensive implementation plan on maternal, infant and young child nutrition: biennial report; 2021.

4. Auerbach M. Anemia in Pregnancy; 2023. Available from: www.uptodate.com.

5. Barut A, Mohamud DO. The association of maternal anaemia with adverse maternal and foetal outcomes in Somali women: a prospective study. BMC Womens Health. 2023;23: 193. doi: [10.1186/s12905-023-02382-4](https://doi.org/10.1186/s12905-023-02382-4).

6. WOMAN-2 trial collaborators. Electronic address: woman2@lshtm.ac.uk, WOMAN-2 trial collaborators. Maternal anaemia and the risk of postpartum haemorrhage: a cohort analysis of data from the WOMAN-2 trial. Lancet Glob Health. 2023;11: e1249–e1259-e59. Epub 20230627. doi: [10.1016/S2214-109X(23)00245-0](https://doi.org/10.1016/s2214-109x(23)00245-0).
