# Supplementary material for: Anemia during pregnancy and adverse maternal outcomes in Georgia–A birth registry-based cohort study
Source: PLoS One. 2025 Jan 30;20(1):e0294832. doi: 10.1371/journal.pone.0294832 (PMC11781653; doi:10.1371/journal.pone.0294832)
Supplement: S1 Table — (DOCX) [file pone.0294832.s003.docx]

Supplementary Table 1. Maternal Baseline Characteristics by Anemia Status for the Analytical Study Sample

| **Characteristics^[[1]](#footnote-1)^** | **No anemia** | **Mild** | **Moderate** | **Severe** | **No Hb measurement** | **All** |
| --- | --- | --- | --- | --- | --- | --- |
| n of cases by anemia status (row, %) | 86,913 (54.8) | 30,686 (19.3) | 11,698 (7.4) | 662 (0.4) | 28,709 (18.1) | 158,668 (100) |
| Prevalence^[[2]](#footnote-2)^ | 86,913 (66.9) | 30,686 (23.6) | 11,698 (9.0) | 662 (0.5) | - | 129,959 (100) |
| Year of delivery, n (row, %)  2019  2020  2021  2022 | 20,952 (46.4)  24,249 (55.2)  26,013 (59.9)  15,699 (60.2) | 9,969 (22.0)  8,409 (19.2)  7,942 (18.3)  4,366 (16.7) | 4,221 (9.3)  3,332 (7.5)  2,650 (6.1)  1,495 (5.7) | 417 (0.9)  196 (0.5)  42 (0.1)  7 (0.1) | 9,687 (21.4)  7,728 (17.6)  6,768 (15.6)  4,526 (17.3) | 45,246 (100)  43,914 (100)  43,415 (100)  26,093 (100) |
| Age, mean (SD) | 28.4 (5.79) | 28.2 (5.79) | 27.9 (5.81) | 28.6 (5.78) | 28.2 (6.13) | 28.3 (5.85) |
| Age groups, n (%)  ≤ 19  20-29  30-34  34-39  ≥40 | 4,285 (4.9)  46,999 (54.1)  21,813 (25.1)  10,813 (12.4)  3,003 (3.5) | 1,672 (5.5)  16,899 (55.1)  7,294 (23.8)  3,854 (12.6)  967 (3.0) | 729 (6.3)  6.520 (55.7)  2,762 (23.6)  1,348 (11.5)  339 (2.9) | 31 (4.7)  358 (54.1)  157 (23.7)  94 (14.2)  22 (3.3) | 1.984 (6.9)  15,133 (52.7)  6,874 (23.9)  3,536 (12.3)  1,182 (4.2) | 8,701 (5.4)  85,909 (54.1)  38,900 (24.5)  19,645 (12.4)  5,513 (3.5) |
| Residency, n (%)  Urban  Rural  Unknown | 64,485 (74.2)  22,420 (25.7)  8 (0.1) | 22,934 (74.6)  7,750 (25.3)  2 (0.1) | 8,598 (73.5)  3,100 (26.5)  0 | 500 (75.5)  162 (24.5)  0 | 19,879 (69.3)  8,800 (30.6)  30 (0.1) | 116,396 (73.4)  42,232 (26.6)  40 (0.03) |
| Education, n (%)  Primary  Secondary  Higher  Unknown | 5,611 (6.5)  35,339 (40.7)  31,328 (36.1)  14,635 (16.7) | 2,047 (6.7)  12,977 (42.3)  10,506 (34.2)  5,156 (16.8) | 975 (8.2)  4,926 (42.2)  3,487 (29.8)  2,310 (19.8) | 31 (4.7)  303 (45.8)  209 (31.5)  119 (18.0) | 2,996 (10.4)  12,022 (41.9)  8,470 (29.5)  5,221 (18.2) | 11,660 (7.4)  65,567 (41.3)  54,000 (34.0)  27,441 (17.3) |
| ANC visits, n (%)  < 4  4-8  >8 | 3,875 (4.4)  66,298 (76.3)  16,740 (19.3) | 895 (2.9)  22,684 (73.9)  7,107 (23.2) | 426 (3.6)  8,380 (71.7)  2,892 (24.7) | 26 (3.9)  446 (67.4)  190 (28.7) | 11,228 (39.1)  16,148 (56.3)  1,333 (4.6) | 16,450 (10.4)  113,956 (71.8)  28,262 (17.8) |
| BMI, n (%)^[[3]](#footnote-3)^  <18.5  18.5-24.9  25-30  >30 | 5,587 (6.6)  49,320 (58.6)  18,759 (22.4)  10,438 (12.4) | 2,237 (7.5)  18,052 (60.8)  6,398 (21.6)  3,008 (10.1) | 886 (7.9)  6,999 (62.4)  2,242 (20.0)  1,094 (9.7) | 48 (7.5)  401 (62.4)  131 (20.4)  63 (9.7) | 1,597 (7.3)  13,278 (60.5)  4,713 (21.4)  2,371 (10.8) | 10,355 (7.0)  88,050 (59.7)  32,243 (21.9)  16,974 (11.5) |
| Parity, n (%)  Nullipara  Multipara | 35,968 (41.4)  50,945 (58.6) | 11,535 (37.6)  19,151 (62.4) | 4,143 (35.4)  7,555 (64.6) | 247 (37.3)  415 (62.7) | 10,308 (35.9)  18,401 (64.1) | 62,201 (39.2)  96,467 (60.8) |
| Plurality, n (%)  Singleton  Multiple | 85,632 (98.5)  1,281 (1.5) | 30,227 (98.5)  459 (1.5) | 11,451 (97.9)  247 (2.1) | 649 (98.0)  13 (2) | 28,086 (97.8)  623 (2.2) | 156,045 (98.4)  2,623 (1.6) |
| GA at lowest Hb value, mean (SD) | 29.8 (5.79) | 30.5 (5.52) | 30.9 (5.81) | 29.2 (6.22) | - | 30.1 (5.75) |
| Lowest recorded Hb value, mean (SD) | 115 (0.79) | 102 (0.34) | 90 (0.74) | 57 (0.79) | - | 109 (1.14) |
| Bleeding during pregnancy, n (%)  Yes  No | 1,263 (1.5)  85,650 (98.5) | 627 (2.0)  30,059 (98.0) | 229 (2.0)  11,469 (98.0) | 12 (1.8)  650 (98.2) | 324 (1.1)  28,385 (98.9) | 2,455 (1.5)  156,213 (98.5) |
| Mode of delivery, n (%)  CS  Vaginal | 36,249 (41.7)  50,663 (58.3) | 12,781 (41.7)  17,902 (58.3) | 5,105 (43.6)  6,593 (56.4) | 273 (41.2)  389 (58.8) | 12,216 (42.5)  16,492 (57.5) | 66,624 (42.0)  92,039 (58.0) |

1. SD – standard deviation; ANC visits – antenatal care visits; BMI – body mass index; GA – gestational age; Hb – hemoglobin; CS – cesarean section

   For defining anemia status (no anemia, mild anemia, moderate anemia, and severe anemia) is used hemoglobin (Hb) cutoffs for pregnant women, considering pregnancy trimester as well, following the Guideline on Hb cutoffs to define anemia in individuals and populations, by the World Health Organization, 2024 [↑](#footnote-ref-1)
2. This number was calculated based on number of women who had at least Hb measurement during the whole pregnancy [↑](#footnote-ref-2)
3. 11,046 women did not have any BMI measures, indicated in the GBR [↑](#footnote-ref-3)
