# Supplementary material for: Anemia during pregnancy and adverse maternal outcomes in Georgia–A birth registry-based cohort study
Source: PLoS One. 2025 Jan 30;20(1):e0294832. doi: 10.1371/journal.pone.0294832 (PMC11781653; doi:10.1371/journal.pone.0294832)
Supplement: S2 Table — (DOCX) [file pone.0294832.s004.docx]

Supplementary Table 2. Regional distribution of performed Hb testes among pregnant women

| **Regions^[[1]](#footnote-1)^** | **At least one Hb test during pregnancy,**  **n (%)** | **No Hb measurement, n (%)** |
| --- | --- | --- |
| Samtskhe-Javakheti | 5,540 (92.9) | 423 (7.1) |
| Racha-Lechkhumi and Kvemo Svaneti | 633 (85.4) | 108 (14.6) |
| Mtskheta-Mtianeti | 2,292 (79.6) | 589 (20.4) |
| Shida Kartli | 8,384 (82.7) | 1,754 (17.3) |
| Kakheti | 8,839 (75.6) | 2,850 (24.4) |
| Tbilisi | 44,684 (82.9) | 9,211 (17.1) |
| Samegrelo and Zemo Svaneti | 6,707 (66.7) | 3,353 (33.3) |
| Guria | 2,891 (85.9) | 474 (14.1) |
| Imereti | 17,182 (92) | 1,502 (8.0) |
| Kvemo Kartli | 14,604 (77.2) | 4,307 (22.8) |
| Adjara | 16,022 (82.2) | 3,471 (17.8) |
| **Georgia (countrywide)** | **129,959 (81.9)** | **28,709 (18.1)** |

1. Excluded pregnancies, delivered in Abkhazia and abroad [↑](#footnote-ref-1)
