# Supplementary material for: Anemia during pregnancy and adverse maternal outcomes in Georgia–A birth registry-based cohort study
Source: PLoS One. 2025 Jan 30;20(1):e0294832. doi: 10.1371/journal.pone.0294832 (PMC11781653; doi:10.1371/journal.pone.0294832)
Supplement: S3 Table — (DOCX) [file pone.0294832.s005.docx]

Supplementary Table 3. Maternal Baseline Characteristics by Antenatal visit’s (ANC) attended pregnant women

| **Characteristics** | **Attending ANC, with Hb measurement** | **Attending ANC, with no Hb measurement** | **No ANC** |
| --- | --- | --- | --- |
| n (row, %) | 129,959 (78.3) | 28,709 (17.3) | 7,375 (4.4) |
| Year of delivery, n (row, %)  2019  2020  2021  2022 | 35,560 (74.9)  36,187 (79.1)  36,645 (80.9)  21,567 (78.3) | 9,686 (20.4)  7,728 (16.9)  6,768 (14.9)  4,526 (16.5) | 2,224 (4.7)  1,830 (4.0)  1,892 (4.2)  1,429 (5.2) |
| Age, mean (SD) | 28.3 (5.8) | 28.2 (6.1) | 28.4 (6.3) |
| Age groups, n (%)  ≤ 19  20-29  30-34  34-39  ≥40 | 6,717 (5.2)  70,779 (54.5)  32,027 (24.6)  16,109 (12.4)  4,221 (3.3) | 1,984 (6.9)  15,133 (52.7)  6,873 (23.9)  3,536 (12.3)  1,182 (4.1) | 524 (7.1)  3,699 (50.2)  1,813 (24.6)  1,018 (13.8)  321 (4.4) |
| Residency, n (%)  Urban  Rural  Unknown | 96,519 (74.2)  33,430 (25.7)  10 (0.1) | 19,879 (69.2)  8,799 (30.7)  30 (0.1) | 5,257 (71.3)  2,039 (27.6)  79 (1.1) |
| Education, n (%)  Primary  Secondary  Higher  Unknown | 8,662 (6.7)  53,547 (41.2)  45,530 (35.0)  22,220 (17.1) | 2,996 (10.4)  12,021 (41.9)  8,470 (29.5)  5,221 (18.2) | 830 (11.3)  2,605 (35.3)  1,360 (18.4)  2,580 (35.0) |
| Parity, n (%)  Nullipara  Multipara | 51,890 (39.9)  78,069 (60.1) | 10,308 (35.9)  18,400 (64.1) | 2,338 (31.7)  5,037 (68.3) |
| Plurality, n (%)  Singleton  Multiple | 127,959 (98.5)  2,000 (1.5) | 28,085 (97.8)  623 (2.2) | 7,274 (98.6)  101 (1.4) |
| Mode of delivery, n (%)  CS  Vaginal | 54,408 (41.9)  75,547 (58.1) | 12,216 (42.6)  16,492 (57.4) | 2,435 (33.0)  4,940 (67.0) |

SD – standard deviation; ANC visits – antenatal care visits; Hb – hemoglobin; CS – cesarean section
