# Supplementary material for: Anemia during pregnancy and adverse maternal outcomes in Georgia–A birth registry-based cohort study
Source: PLoS One. 2025 Jan 30;20(1):e0294832. doi: 10.1371/journal.pone.0294832 (PMC11781653; doi:10.1371/journal.pone.0294832)
Supplement: S4 Table — (DOCX) [file pone.0294832.s006.docx]

Supplementary Table 4. Antenatal care attendance rate per Georgian regions during the study period

| **Regions** | **No attendance, %** | **At least one visit, %** |
| --- | --- | --- |
| Adjara | 2.6 | 97.4 |
| Imereti | 2.6 | 97.4 |
| Samtskhe-Jvakheti | 2.6 | 97.4 |
| Racha | 2.8 | 97.2 |
| Shida Kartli | 2.9 | 97.1 |
| Guria | 3.2 | 96.8 |
| Tbilisi | 4.8 | 95.2 |
| Kvemo Kartli | 5.2 | 94.8 |
| Samegrelo | 5.4 | 94.6 |
| Kakheti | 6.1 | 93.9 |
| Mtskheta-Mtianeti | 6.6 | 93.4 |
